# Construction of an efficient *Claviceps paspali* cell factory for lysergic acid production

**DOI:** 10.3389/fbioe.2022.1093402

**Published:** 2023-01-25

**Authors:** Mingzhe Hu, Yu Zhou, Siyu Du, Xuan Zhang, Shen Tang, Yong Yang, Wei Zhang, Shaoxin Chen, Xuenian Huang, Xuefeng Lu

**Affiliations:** ^1^ College of Life Sciences, Qingdao University, Qingdao, China; ^2^ Shandong Provincial Key Laboratory of Synthetic Biology, Qingdao Institute of Bioenergy and Bioprocess Technology, Chinese Academy of Sciences, Qingdao, China; ^3^ Shandong Energy Institute, Qingdao, China; ^4^ Qingdao New Energy Shandong Laboratory, Qingdao, China; ^5^ Institute for Smart Materials and Engineering, University of Jinan, Jinan, China; ^6^ Shisenhai (Hangzhou) Biopharmaceutical Co., Ltd., Hangzhou, China; ^7^ University of Chinese Academy of Sciences, Beijing, China; ^8^ State Key Lab of New Drug and Pharmaceutical Process, Shanghai Institute of Pharmaceutical Industry, Shanghai, China; ^9^ Marine Biology and Biotechnology Laboratory, Qingdao National Laboratory for Marine Science and Technology, Qingdao, China

**Keywords:** lysergic acid, *Claviceps paspali*, metabolic engineering, medium composition, fermentation optimization

## Abstract

Lysergic acid (LA) is the key precursor of ergot alkaloids, and its derivatives have been used extensively for the treatment of neurological disorders. However, the poor fermentation efficiency limited its industrial application. At the same time, the hardship of genetic manipulation has hindered the metabolic engineering of *Claviceps* strains to improve the LA titer further. In this study, an efficient genetic manipulation system based on the protoplast-mediated transformation was established in the industrial strain *Claviceps paspali*. On this basis, the gene *lpsB* located in the ergot alkaloids biosynthetic gene cluster was deleted to construct the LA-producing cell factory. Plackett-Burman and Box-Behnken designs were used in shaking flasks, achieving an optimal fermentation medium composition. The final titer of LA and iso-lysergic acid (ILA) reached 3.7 g·L^−1^, which was 4.6 times higher than that in the initial medium. Our work provides an efficient strategy for the biosynthesis of LA and ILA and lays the groundwork for its industrial production.

## 1 Introduction

Ergot alkaloids (EA) are a class of natural products known for their extensive pharmacologic activities (Wallwey and Li, 2011; Hulvová et al., 2013; Jakubczyk et al., 2014). They are structurally similar to dopamine, serotonin and adrenaline, and exhibit neurotoxicity by acting on the neurotransmitter receptors of tryptamine derivatives (Mantegani et al., 1999; Liu and Jia, 2017). Many natural and semi-synthetic ergot alkaloids, such as ergometrine, nicergoline and cabergoline, have been developed as important clinical drugs to treat *postpartum* hemorrhages, Alzheimer’s disease, Parkinson’s disease and other disorders (Baskys and Hou, 2007; Perez-Lloret and Rascol, 2010; Robinson and Panaccione, 2015). As an important pharmaceutical intermediate, LA is applied to the industrial production of nicergoline, cabergoline and dihydroergotamine. And LA derivatives, such as lysergic acid diethylamide (LSD), have been widely used in the therapy of psychiatric diseases (Young et al., 2015; Chen et al., 2017). Due to high demand in the market, up to 10–15 tons of LA have been produced annually (Hulvová et al., 2013; Wong et al., 2022). However, the current industrial production of LA shows low production efficiency and high cost. There is no sophisticated scheme to directly produce LA in the current industry. All the LA is acquired from alkali hydrolysis of ergometrine. About 40%–50% of ergometrine is obtained from the parasitic production of ergot on rye (field cultivation), which highly depends on the climate and growth conditions of the host (Tudzynski et al., 2001). The rest of ergometrine has been contributed by the submerged fermentation using *Claviceps paspali* (Hulvová et al., 2013; Wong et al., 2022). Complicated post-processing and separation severely restrict the yield. Hence, efficient LA-producing cell factories are urgently needed to improve the production efficiency in industry.

**FIGURE 1:**
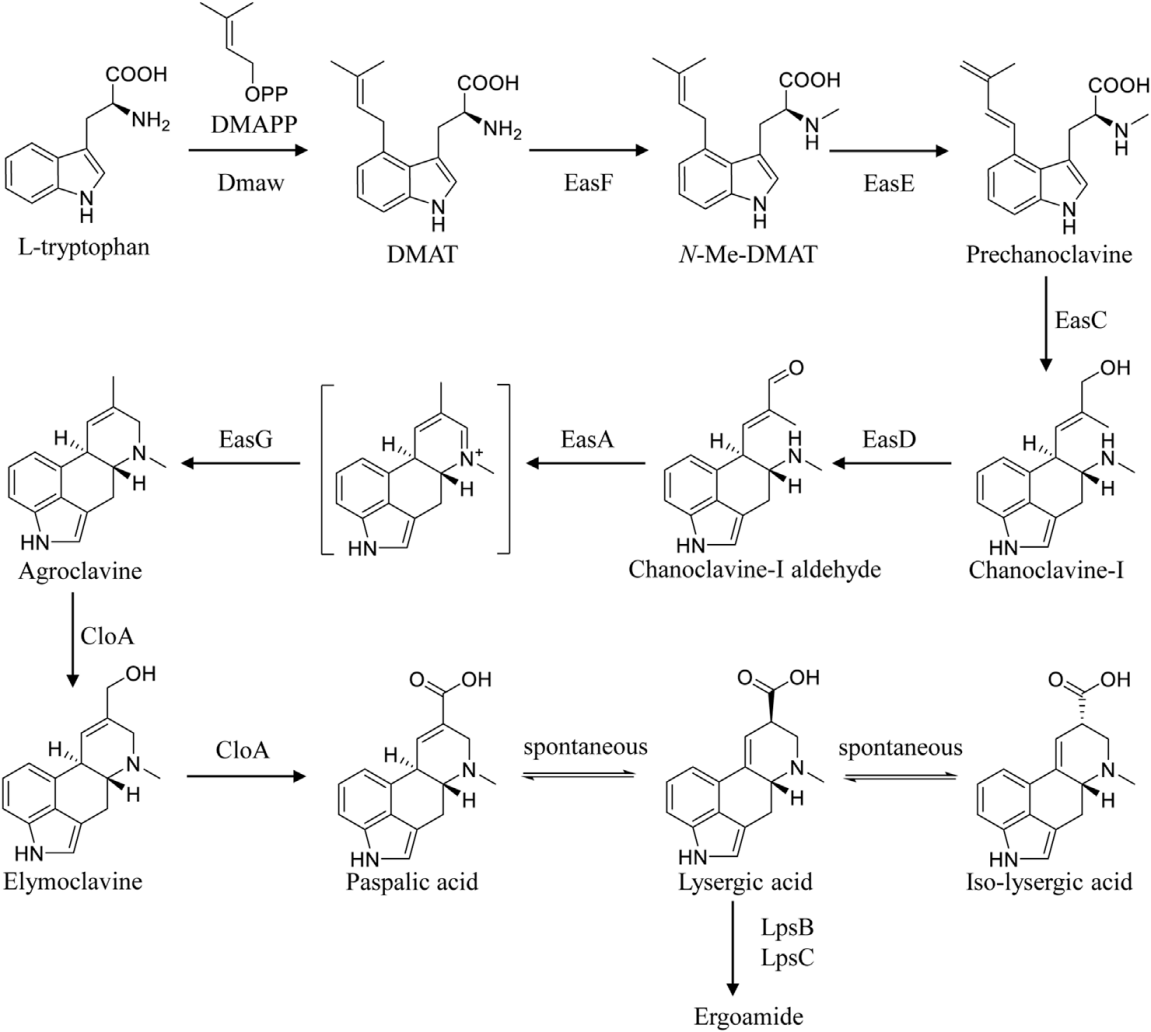
The complete biosynthetic and metabolic pathways of LA in *C. paspali.*

In *Claviceps*, the biosynthetic pathway of EA is broadly classified into three parts (Chen et al., 2017). Firstly, the biosynthesis of chanoclavine-I-aldehyde, the common steps in all EA-producing species, requires five enzymes including DmaW, EasF, EasC, EasE, and EasD (Chen et al., 2017; Wong et al., 2022). Then, the introduction of EasA, EasG, and CloA can result in the formation of LA from chanoclavine-I-aldehyde (Robinson and Panaccione, 2014; Chen et al., 2017). Finally, LA is activated by LpsB and converted into ergoamides by LpsC (Correia et al., 2003; Ortel and Keller, 2009; Chen et al., 2017). Progress in ergot alkaloid biosynthesis provides new clues for the construction of LA-producing cell factories (Figure 1)(Chen et al., 2017). Wong *et al.* reconstituted the LA biosynthetic pathway in *Saccharomyces cerevisiae* and screened the key enzymes for functional expression from different sources, constructing a LA-producing strain with 1.7 mg·L^−1^ titer after 5 days culture in a 1 L bioreactor (Wong et al., 2022). Yao *et al.* expressed the key genes involved in EA biosynthesis in heterologous host *Aspergillus nidulans*, yielding trace amounts of LA (Yao et al., 2022). Besides, other engineered heterologous LA-producing strains, for instance, *Metarhizium brunneum*, *Neosartorya fumigate*, and *Aspergillus fumigatus*, were constructed with low titer (Robinson and Panaccione, 2014; Arnold and Panaccione, 2017; Davis et al., 2020; Yao et al., 2022). However, LA production in the above heterogeneous cell factories cannot meet actual industrial demand. It has been reported that some of the enzymes involved in the biosynthetic pathway of EA were poorly expressed in heterologous host (Coyle et al., 2010; Nielsen et al., 2014; Wong et al., 2022), hampering the improvement of the production. Thus, we turn to the metabolic engineering of industrial strains *C. paspali* MJXA-WT with high-yield ergometrine, which may avoid the incompatibility of expression elements and heterologous host. The genetic transformation method for the genus *Claviceps* has been reported over the past decades (Hulvová et al., 2013). Protoplast-mediated transformation is the most popular method for the genetic transformation of *Claviceps*. A successful transformation method for *Claviceps purpurea* based on the protoplast preparation was first described in 1989 (van Engelenburg et al., 1989). And the conditions of protoplast formation and regeneration were further optimized (Comino et al., 1989; Smit and Tudzynski, 1992; Mey et al., 2002; Wang et al., 2016). Besides, the *Agrobacterium tumefaciens*-mediated transformation method for *C. purpurea* has been attempted, but no successful procedure has been described (Hulvová et al., 2013). However, there are few reports on the genetic transformation for *C. paspali*. Engelenburg *et al.* developed a protoplast-mediated transformation system for *C. purpurea*, while suffering from low protoplast regeneration efficiency when applied to *C. paspali* (van Engelenburg et al., 1989; Kozák et al., 2018). The *Agrobacterium tumefaciens*-mediated transformation system had been successfully applied in *C. paspali*, which is operational complexity and time consuming (Kozák et al., 2018).

The lack of genetic tools of *C. paspali* hindered the process of constructing high-yield strains through metabolic engineering. In this study, we established an effective system of genetic manipulation in the industrial strain of *C. paspali* MJXA-WT in the first step. Based on this, the *lpsB* gene was deleted in *C. paspali*, leading to the accumulation of LA and ILA in mutants. Finally, the fermentation process was developed and optimized for the engineered strain in shaking flasks.

## 2 Materials and methods

### 2.1 Microorganism and culture conditions

*C. paspali* MJXA-WT is an industrial ergometrine-producing strain frozen at −80°C in our lab at Qingdao Institute of Bioenergy and Bioprocess Technology, Chinese Academic of Science, China. The strains were maintained on PDA (39 g·L^−1^ PDA dry powder, BD company) at 25°C for 6 days, and then 3 cm^2^ fresh mycelium from the PDA plates was transferred to a 250-mL flask containing 25 mL of seed medium (20 g·L^−1^ mannitol, 10 g·L^−1^ succinic acid, 2 g·L^−1^ soybean cake powder, 1 g·L^−1^ KH_2_PO_4_, 0.3 g·L^−1^ MgSO_4_·7H_2_O, and pH 5.0) and cultured for 4 days at 25°C, 220 rpm. The seed liquid was inoculated into 250-mL flask containing 25 mL of fermentation medium (100 g·L^−1^ sorbitol, 35 g·L^−1^ succinic acid, 20 g·L^−1^ corn steep powder, 0.5 g·L^−1^ yeast extract powder, 0.022 g·L^−1^ FeSO_4_·7H_2_O, 0.01 g·L^−1^ ZnSO_4_·7H_2_O, 0.7 g·L^−1^ MgSO_4_·7H_2_O, pH 5.5). The flasks were kept in the dark for 12 days at 25°C, 220 rpm. An inoculum ratio of 15% (v/v) was used in all bioprocess. All the experiments were repeated three times in this study.

### 2.2 DNA manipulation for cassettes construction

Primers used in this study are listed in Supplementary Table S1. To knock out gene *lpsB*, primer pairs Up-lpsB-F/Up-lpsB-hph-R and Down-lpsB-hph-F/Down-lpsB-R were used to amplify the flanking 5' and 3' DNA of the *lpsB* gene from the genome of *C. paspali* MJXA-WT. The resulting DNA fragments were fused with the *hph* marker by fusion PCR. Primers N-lpsB-F/N-lpsB-R were used to amplify the gene-targeting cassette.

### 2.3 Protoplast-mediated transformation

About 3 cm^2^ of fresh mycelium from the PDA plates was crushed and inoculated into 100 mL of PDB medium and cultured for 3–4 days at 25°C, 220 rpm. The mycelium was collected by filtering with sterile 300 mesh nylon cloth, and washed twice with 0.7 M KCl solution. The mycelium was immersed in the enzymatic solution (0.1% of lywallzyme), and incubated at 30°C for 1 h to prepare protoplasts. The culture solution was filtered with 500 mesh nylon cloth and centrifuged for 12 min at 4,000 rpm to obtain protoplasts. The protoplasts were resuspended with 10 mL of STC solution (0.85 M sorbitol, 10 Tris-HCl pH 8.0, 50 mM CaCl_2_), and centrifuged for 10 min at 3,400 rpm. The precipitation of protoplasts was diluted to 10^7^ cells/mL. Then 2–4 μg of DNA fragments, 100 μL protoplast solution and 50 μL of ice-cold PSTC (25% PEG 6000, 1 M D-sorbitol, 10 mM Tris-HCl pH 8.0, and 50 mM CaCl_2_) were mixed, and incubated on ice for 25 min. After that, 1 mL of PSTC was added and incubated for 20 min. Afterwards, 20 mL of liquid top agar (PDB with 0.5% agarose and 0.4 M D-sorbitol) was added and the mixture was spread on PDAS (PDA with 0.4 M D-sorbitol). The plates were cultured in the dark for 7 days at 30 C.

### 2.4 HPLC analysis

LA and ILA were analyzed by high-performance liquid chromatography (HPLC) equipped with a C18 reversed-phase column (Agilent, 4.6 × 150 mm, 5 μm). 1 mL of fermentation supernatant was extracted with 4 mL of mixed solvent containing acetonitrile and water (1:1, v/v) for HPLC and LC-MS measurement. The mobile phases were as follows: water with 0.1% (NH_4_)_2_CO_3_ (solvent A); and 75% acetonitrile (solvent B). Chromatography was carried out over a flow rate of 1.2 mL/min, with a stepped gradient as follows: 13%–43% B from 0–12 min, 43% B for 1 min, 13% B for 1 min. LA and ILA were quantified by comparing peak areas to standard curves under 254 nm. The titers of LA and ILA were quantified by HPLC according to the calibration curves respectively. The calibration curves were presented as Supplementary Figure S1.

### 2.5 Plackett-Burman design (PBD) of the experiment

To determine the significant factors influencing the LA and ILA titer in submerged fermentation, six factors including inoculum amount (A), succinic acid (B), corn steep powder (C), yeast extract (D), sorbitol (E), and MgSO_4_·7H_2_O (F) were handpicked for PBD. The final titer of total lysergic acid (TLA), including LA and ILA, was chosen as the response. For each independent factor, there were high (+1) and low (−1) levels, which were determined based on the single factor experiment, and used to assess the influence of each factor. The coded level and actual level of each factor are listed in Table 1. All experiments were repeated three times.

**TABLE 1:** PBD for the evaluation of the factors influencing TLA titer.

Code factors	Factors	Coded level and actual level	
		−1	+1
A	Inoculum amount (mL)	3	5
B	Succinic acid (g·L^−1^)	25	45
C	Corn steep powder (g·L^−1^)	10	30
D	Yeast extract (g·L^−1^)	0	0.5
E	Sorbitol (g·L^−1^)	100	140
F	MgSO_4_·7H_2_O (g·L^−1^)	1	2

### 2.6 Box-Behnken design (BBD) of the experiment

The most monumental factors were chosen to identify their optimal level with the LA and ILA titer as the response. Three significant factors identified by PBD were corn steep powder (A), succinic acid (B) and yeast extract (C). For each variable, there were low (−1), middle (0) and high (+1) levels. The coded level and actual level of three factors is listed in Table 2. All the experiments were repeated three times.

**TABLE 2:** BBD for the evaluation of the factors influencing TLA titer.

Code factors	Factors	Coded level and actual level		
		-1	0	+1
A	Corn steep powder (g·L^−1^)	10	20	30
B	Succinic acid (g·L^−1^)	25	35	45
C	Yeast extract (g·L^−1^)	0	0.25	0.5

## 3 Results and discussion

### 3.1 Development of genetic manipulation system in *C. paspali*

**FIGURE 2:**
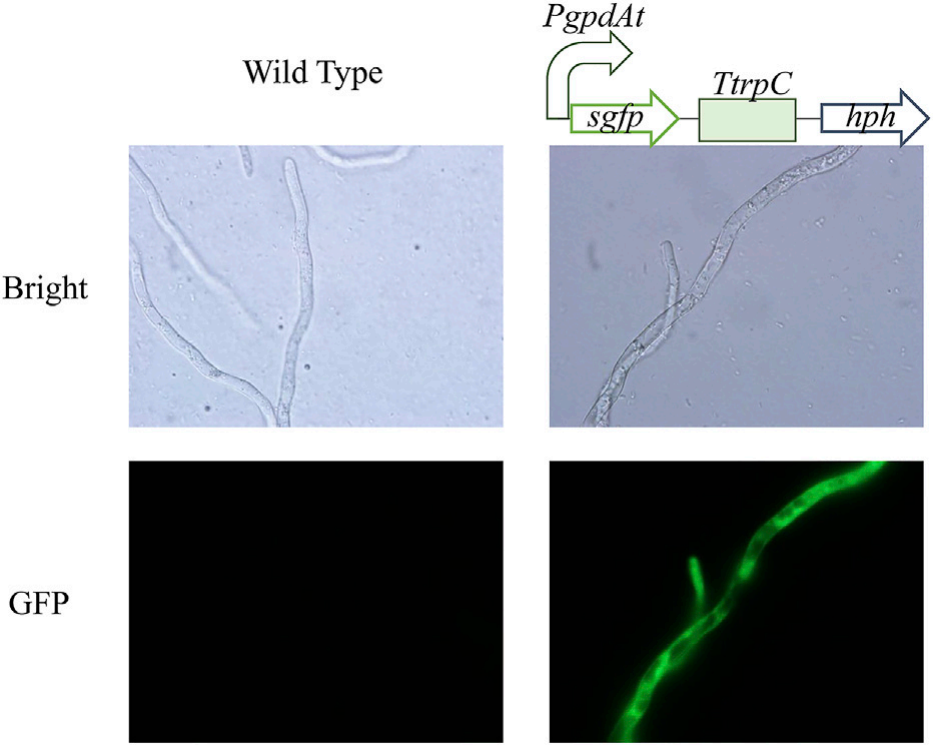
Microscopic features of wild-type and mutant strains. Bright: Bright-field images. GFP: Fluorescence images.

Inefficient genetic manipulation system restricted metabolic engineering of *C. paspali*. Protoplast-mediated transformation is widely and efficiently used in ascomycetes transformation. Therefore, we choose this method to establish the genetic manipulation system in *C. paspali*. As an industrial strain, *C. paspali* MJXA-WT lost the sporulation ability, the mycelium from PDA plates was used as seeds for cultivation of mycelium. Poor mycelium leads to difficulties in protoplast preparation and low efficiency of cell wall regeneration. Homologous recombination usually occurs in logarithmic growth period. The regeneration efficiency was mainly depended on the culture time of mycelia. We tried to apply the mycelia of non-sporulating *C. paspali* MJXA-WT cultured in original medium for different time to transformation. However, it showed very low regeneration efficiency probably due to the slow growth state. Luckily, we found that the mycelia of *C. paspali* MJXA-WT grew very fast only in the PDB medium, and the hyphae of *C. paspali* MJXA-WT cultivated in PDB medium for 3-days was applied to the enzymatic digestion in next step. Enzyme solution of 0.1% lywallzyme was used to digest cell wall, which produced 1.0 × 10^6^ cells/mL at 30°C in an hour or so. STC solution was used to wash and resuspend the protoplasts at 1.0 × 10^7^ cells/mL. In the PEG-CaCl_2_ mediated transformation, 2–4 μg of DNA fragments, a cassette containing *PgpdAt* (constitutive promoter of glyceraldehyde-3-phosphate dehydrogenase from *Aspergillus terreus*), *sgfp* (synthetic green fluorescent protein), *TtrpC* (terminator of tryptophan synthetase from *Aspergillus nidulans*) and *hph* (hygromycin B phosphotransferase gene), was added in 100 μL protoplasts solution. The D-sorbitol concentration of regeneration medium was optimized, and the optimal concentration was 0.4 M (Supplementary Table S2). Beginning with 1.0 × 10^7^ protoplasts, approximately 120–150 positive clones were obtained in a reaction (Supplementary Figure S2). Transformants were verified by genomic PCR. The representative mutant strains displayed significant green fluorescence under the fluorescence microscope, while the parental strain did not show any visible fluorescence (Figure 2). Consequently, an efficient genetic manipulation system, which was based on the protoplast-mediated transformation, was established in the industrial strain *C. paspali* MJXA-WT.

### 3.2 Construction of the LA-producing cell factory

**FIGURE 3:**
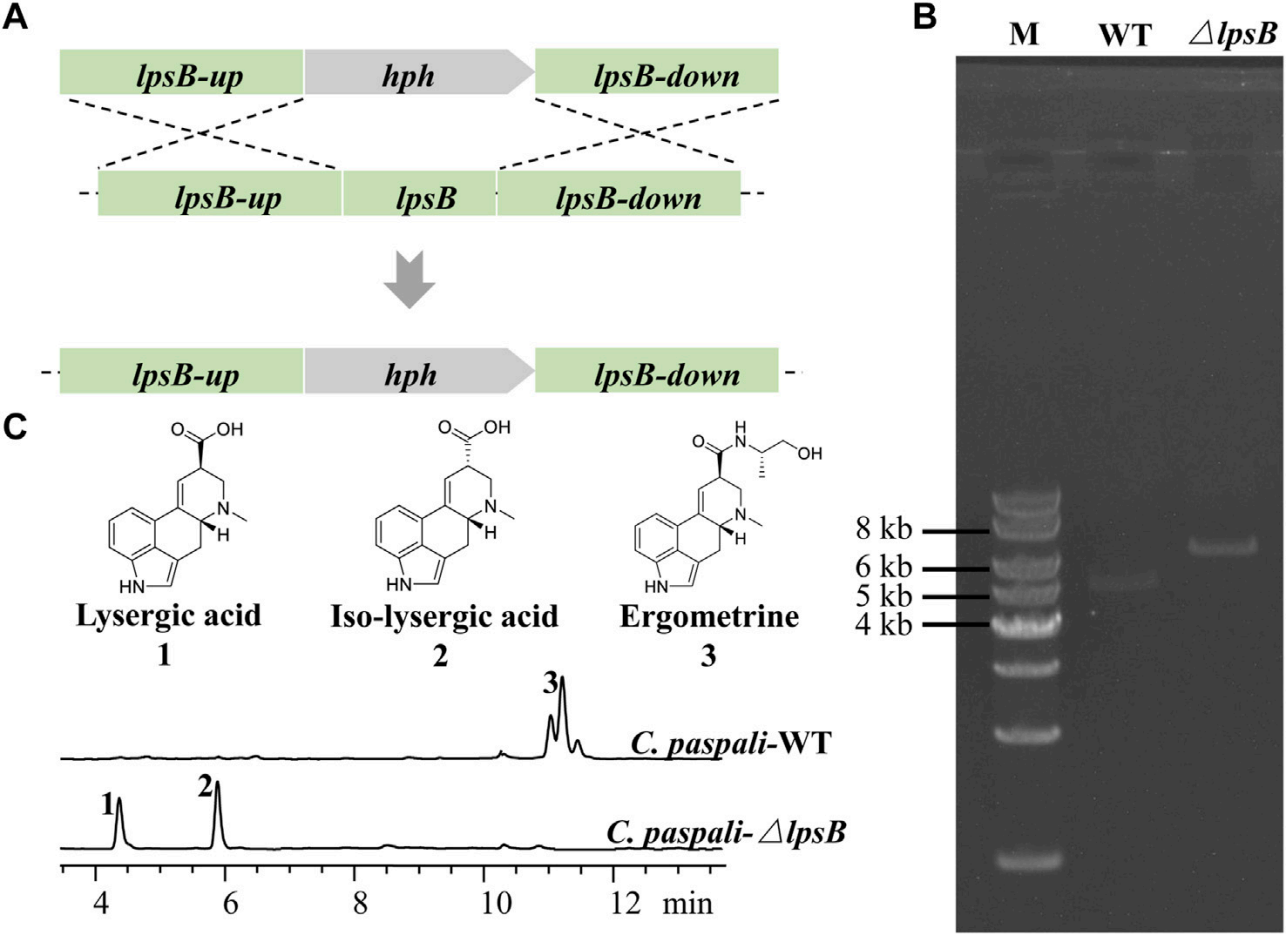
Construction of the LA-producing cell factory. **(A)** Strategy for deleting *lpsB* gene in *C. paspali* MJXA-WT. **(B)** Genotype verification. Lane M: 1 kb DNA marker; lane WT: *C. paspali* MJXA-WT; lane *ΔlpsB*: parent strain *C. paspali* MJXA-*ΔlpsB-hph*. **(C)** HPLC analysis of wild-type and mutant strains.

The gene *lpsB* was involved in the modification of LA in the biosynthetic pathway of ergometrine (Figure 1). We tried to knock out *lpsB* to prevent the biosynthesis of ergometrine, resulting in the accumulation of LA. The targeting element was constructed using *hph* as a marker. About 1 kb region in *lpsB* gene was replaced by the *hph* (2.2 kb) (Figure 3A). As shown in Figure 3B, the *lpsB* gene was successfully disrupted in mutant strains (Figure 3B). Knocking out *lpsB* eliminated the production of ergometrine but accumulated two new products with identical molecular ion peaks ([M + H]^+^ 269.1303) (Figure 3C; Supplementary Figure S3). The new products were identified as LA and ILA by further NMR analyses (Supplementary Figures S4, 5) (Liu and Jia, 2011). The ^1^H and ^13^C NMR data of ILA showed quite similar signals to those of LA. The noticeable differences were the occurrence of C-8 (δC 42.7, δH 3.54–3.50) in ILA, rather than C-8 (δC 44.4, δH 4.09–4.04) carbons in LA. Supplementary Table S3 indicated the attribution of ^1^H and ^13^C NMR data for LA and ILA. Due to its instability, LA could be partially converted to its isomer ILA (Stoll et al., 1949; Himmelsbach et al., 2014; Yao et al., 2022). The percentages of LA were stabilized at around 45%, while the percentages of ILA were stabilized at around 55%. Base on this, we used the titer of TLA to evaluate the change of production in the next step.

### 3.3 Effects of initial media components on LA and ILA accumulation

**FIGURE 4:**
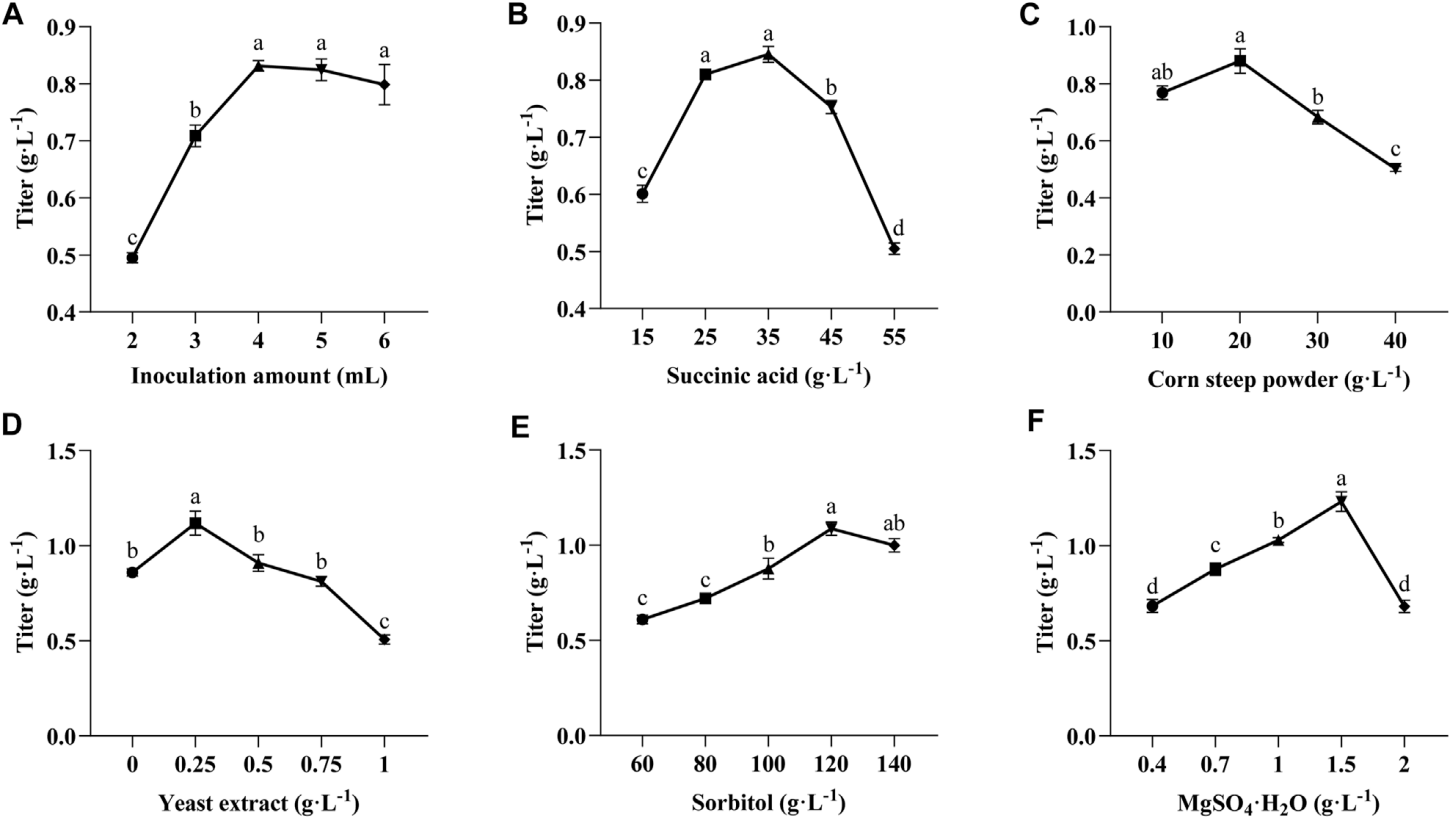
Effect of inoculation amount **(A)** and initial medium components of succinic acid **(B)**, corn steep powder **(C)**, yeast extract **(D)**, sorbitol **(E)** and MgSO_4_·7H_2_O **(F)** on LA and ILA accumulation. Lowercase alphabets indicate that the values are statistically significant with corresponding *p*-values less than 0.05.

Since various factors in the fermentation medium affect the titer of TLA, an effective experimental approach, such as Plackett-Burman and Box-Behnken design, is essential to achieve the optimal production. First, single-factor experiments were selected to assess the influence of inoculum amount and five key components of the medium (succinic acid, corn steep powder, yeast extract, sorbitol, and MgSO_4_·7H_2_O) on the titer of TLA. The influence of different inoculum amount on the titer of TLA was shown in Figure 4A. With the increase of inoculum amount, the titer of TLA improved. The highest titre was obtained when the inoculum amount was 4 mL, reaching 0.83 g·L^−1^. The production curve of the titer of TLA tended to flatten when the inoculum amount was more than 4 mL. The effect of succinic acid with the addition between 25 g·L^−1^ to 45 g·L^−1^ was not significant (Figure 4B), the production was highest when the concentration was 35 g·L^−1^. A high titer of TLA was obtained with 20 g·L^−1^ corn steep powder added, while the titer of TLA decreased when the corn steep powder concentration was further increased (Figure 4C). When the concentration of yeast extract was 0.25 g·L^−1^, the titer of TLA reached the maximum, and then decreased significantly with the increase of yeast extract content (Figure 4D). Increasing sorbitol and MgSO_4_·7H_2_O concentrations enhanced the titer of TLA. A maximum titer of TLA of 1.09 g·L^−1^ and 1.23 g·L^−1^ was achieved when sorbitol and MgSO_4_·7H_2_O were added in the medium with the concentration of 120 g·L^−1^ and 1.5 g L^−1^, respectively (Figures 4E, F).

### 3.4 Identification of significant factors using PBD

PBD, a two-level experimental design (Abdel-Fattah et al., 2005; Abuhena et al., 2022), was further used to screen out the crucial factors which have a significant impact on the titer of TLA. The experimental data were shown in Table 3. And analysis of experimental results was listed in Table 4 using Design Expert 12.0 software. As the F-value was 27.85 and the *p*-value of 0.0011 was less than 0.05, the model was significant. Two factors, succinic acid and yeast extract were identified as the significant factors influencing the titer of TLA, with corresponding *p*-values less than 0.05. The corresponding *p*-values of corn steep powder was less than 0.001, implying that it was highly significant. Others factors, inoculum amount, sorbitol and MgSO_4_·7H_2_O, with corresponding *p*-values more than 0.1, implied that they were not significant. Another important evaluation indicator of the model quality is R^2^. The values of R^2^, predicated R^2^ and adjusted R^2^ were 0.9709, 0.9361, and 0.8327, respectively, indicating that the predicted model was highly fit and effective. The final equation in terms of actual factors was derived as follows:

Y=1.903+0.009A+0.256B−0.363C+0.218D+0.051E−0.006F

**TABLE 3:** PBD factors affecting the titer of TLA.

Run order	A	B	C	D	E	F	Response
	Inoculum size	Succinic acid	Corn steep powder	Yeast extract	Sorbitol	MgSO_4_·7H_2_O	Titer of TLA (g·L^-1^)
1	1	−1	1	1	−1	−1	1.36 ± 0.05
2	1	−1	1	1	−1	1	1.56 ± 0.01
3	−1	−1	−1	1	1	−1	2.19 ± 0.10
4	−1	−1	1	−1	1	1	1.08 ± 0.04
5	1	1	−1	1	1	−1	2.94 ± 0.05
6	1	1	−1	−1	−1	1	2.20 ± 0.01
7	−1	1	−1	1	−1	1	2.57 ± 0.02
8	1	−1	−1	−1	1	1	1.87 ± 0.02
9	−1	1	1	−1	−1	−1	1.60 ± 0.03
10	1	1	1	−1	1	−1	1.54 ± 0.01
11	−1	−1	−1	−1	−1	−1	1.82 ± 0.01
12	−1	1	1	1	1	1	2.10 ± 0.06

**TABLE 4:** ANOVA of PBD with F and *p* values.

Source	Sum of squares	Degrees of freedom	Mean square	F-value	*p*-value
Model	2.9624	6	0.4937	27.85	0.0011
A-Inoculum amount	0.0010	1	0.0010	0.06	0.8210
B-Succinic acid	0.7854	1	0.7854	44.30	0.0012
C-Corn steep powder	1.5769	1	1.5769	88.95	0.0002
D-Yeast extract	0.5677	1	0.5677	32.02	0.0024
E-Sorbitol	0.0310	1	0.0310	1.75	0.2433
F-MgSO_4_·7H_2_O	0.0004	1	0.0004	0.02	0.8853
Residual	0.0886	5	0.0177		
Total	3.0510	11			

According to the regression coefficient, four factors (inoculum amount, succinic acid, yeast extract and sorbitol) showed positive effects on the titer of TLA. Besides, succinic acid and MgSO_4_·7H_2_O showed negative effects. Succinic acid, corn steep powder, and yeast extract were chosen as key factors for further optimization. Succinic acid in the tricarboxylic acid cycle was usually used as a carbon source, which may promote the high level of oxidative metabolism in the *Claviceps* cells, promoting the biosynthesis of secondary metabolites (Hulvová et al., 2013). Fermentation nitrogen (corn steep powder and yeast extract) are particularly rich in protein and amino acid, such as tryptophan and methionine, which may promote the synthesis of ergot alkaloids (Hulvová et al., 2013; Chen et al., 2017).

### 3.5 Optimization of culture medium using BBD

BBD is a reliable method which is usually used to identify the optimum response region for significant factors (Hegazy et al., 2022). In this study, three significant factors (corn steep powder, (A); succinic acid, (B); yeast extract, (C)) were further explored at three levels. The experimental data were shown in Table 5. And analysis of experimental results was listed in Table 6 using Design Expert 12.0 software. As the F-value was 271.56 and the *p*-value was less than 0.0001, the model was highly significant. The corresponding *p*-values of A, B, and C were both less than 0.0001, suggesting that these factors were highly significant. In the same light, the corresponding *p*-values of A^2^, B^2^, and C^2^ were both less than 0.0001. The values of R^2^, predicated R^2^ and adjusted R^2^ were 0.9971, 0.9935, and 0.9731, respectively, indicating that the model was effective and reliable. To predict the optimal point, the final equation fitted to the experimental response results was represented below:

$$Y=3.38+0.36A+0.301B+0.414C+0.055AB+0.135AC+0.113BC-0.703A^{2}-0.615B^{2}-0.525C^{2}$$

**TABLE 5:** BBD for factors affecting the titer of TLA.

Run order	A	B	C	Response
	Corn steep powder	Succinic acid	Yeast extract	Titer of TLA (g·L^-1^)
1	1	0	1	3.09 ± 0.00
2	−1	−1	0	1.49 ± 0.00
3	0	0	0	3.41 ± 0.00
4	−1	0	−1	1.48 ± 0.02
5	1	−1	0	2.02 ± 0.01
6	0	0	0	3.34 ± 0.04
7	0	1	−1	2.00 ± 0.03
8	0	−1	−1	1.63 ± 0.03
9	0	0	0	3.03 ± 0.13
10	1	1	0	2.74 ± 0.00
11	0	−1	1	2.25 ± 0.00
12	0	0	0	3.36 ± 0.00
13	0	1	1	3.07 ± 0.01
14	−1	1	0	1.99 ± 0.07
15	0	0	0	3.44 ± 0.05
16	−1	0	1	2.02 ± 0.03
17	1	0	−1	2.01 ± 0.02

**TABLE 6:** Analysis of variance for regression model of LA and ILA production.

Source	Sum of squares	Degrees of freedom	Mean square	F-value	*p*-value
Model	8.66	9	0.9623	271.56	<0.0001
A	1.04	1	1.04	292.59	<0.0001
B	0.726	1	0.726	204.88	<0.0001
C	1.37	1	1.37	386.48	<0.0001
AB	0.0121	1	0.0121	3.41	0.1071
AC	0.0729	1	0.0729	20.57	0.0027
BC	0.0506	1	0.0506	14.29	0.0069
A^2^	2.08	1	2.08	586.81	<0.0001
B^2^	1.59	1	1.59	449.78	<0.0001
C^2^	1.16	1	1.16	327.81	<0.0001
Residual	0.0248	7	0.0035		
Lack of Fit	0.0135	3	0.0045	1.6	0.3227
Pure Error	0.0113	4	0.0028		
Cor Total	8.69	16			

According to the equation, the predicted optimum of the cultural medium consisted of 37.2 g·L^−1^ succinic acid, 23.5 g·L^−1^ corn steep liquor powder, and 0.4 g·L^−1^ yeast extract powder, and the predicated titer was 3.6 g·L^−1^. The value of R^2^ was 0.997, indicating a great degree of correlation between the experimental and the predicted values.

**FIGURE 5:**
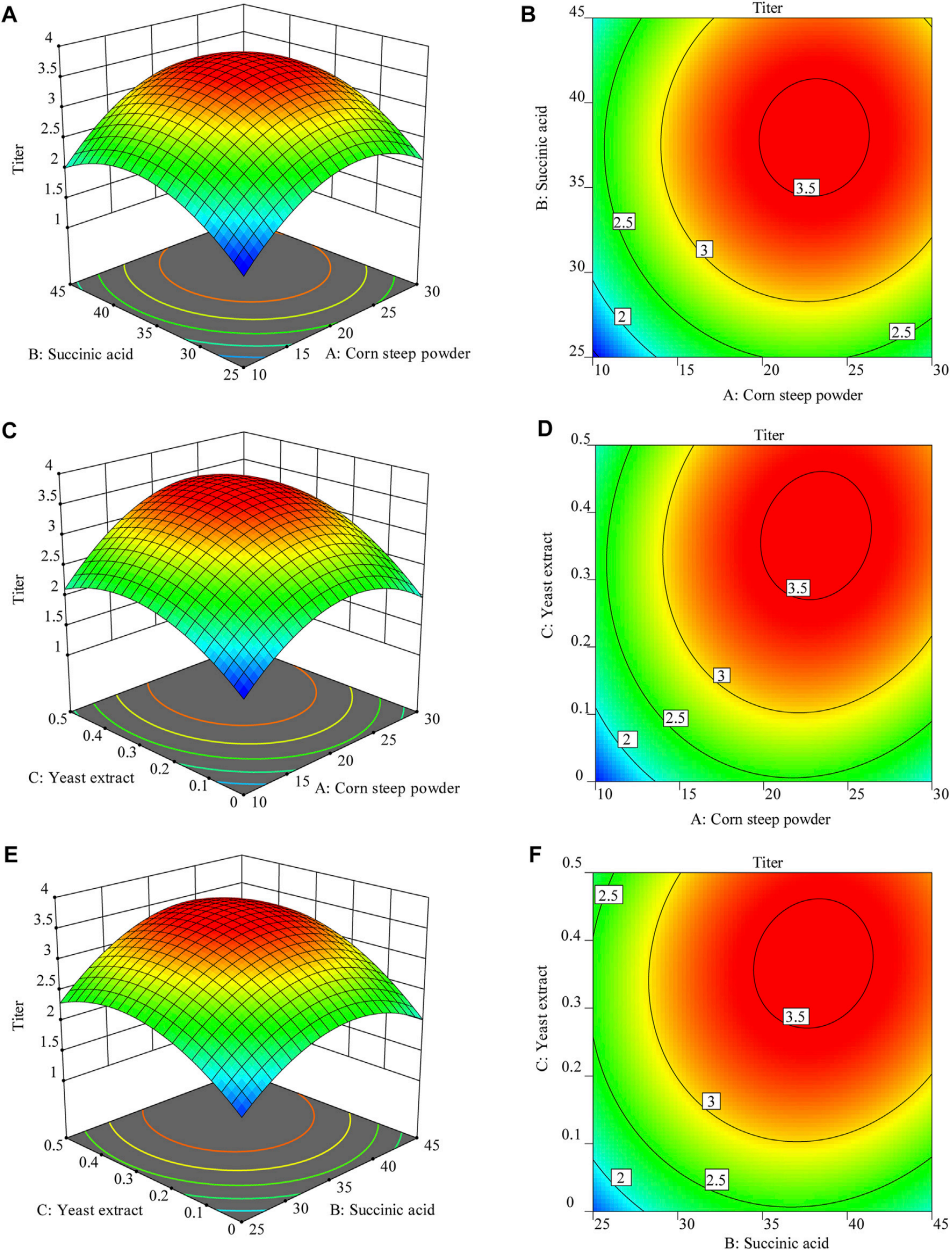
Three-dimensional response surfaces of **(A)** corn steep powder and succinic acid; **(C)** corn steep powder and yeast extract; **(E)** succinic acid and yeast extract, contour plots of **(B)** corn steep powder and succinic acid; **(D)** corn steep powder and yeast extract; **(F)** succinic acid and yeast extract.

To better evaluate the interaction effect between three key factors, three-dimensional response surfaces and contour plots were created in Figure 5. Based on the surface drawing and contour drawing of response surface model, Figures 5A, B showed the interaction effect of corn steep powder and succinic acid on the titer of TLA with the concentration of yeast extract was 0.4 g·L^-1^. Increasing the corn steep powder and succinic acid to the optimal point improved the titer of TLA. However, the increased amount of both factors leaded to the decline of the titer. Figures 5C, D showed the interaction effect of corn steep powder and yeast extract on the titer of TLA with the concentration of succinic acid was 37.2 g·L^−1^. Increasing the addition of corn steep powder and yeast extract to the optimal point improved the titer of TLA. However, improving concentration of both factors leaded to the decline of the titer. Figures 5E, F showed the interaction effect of succinic acid and yeast extract on the titer of TLA with the concentration of corn steep powder was 23.5 g·L^−1^. Increasing the concentration of succinic acid and yeast extract to the optimal point improved the titer of TLA. However, improving concentration of both factors leaded to the decline of the titer.

**FIGURE 6:**
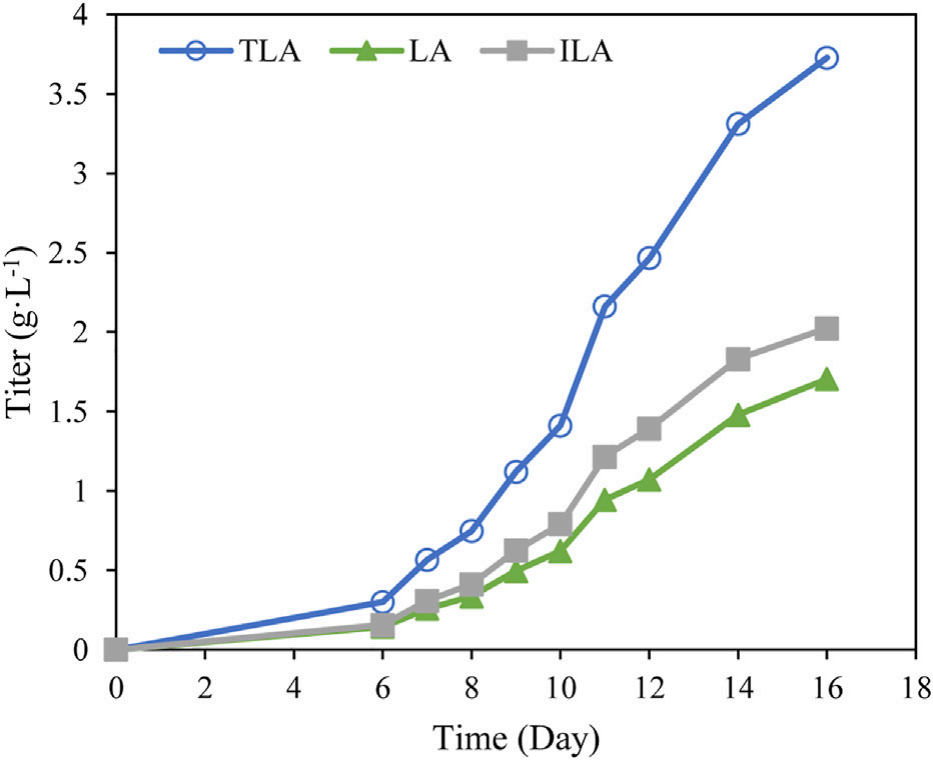
The time profile of the production of TLA using the optimal fermentation medium.

To verify the developed model, the time profile of TLA production under the optimal fermentation condition was analyzed. After 16 days culture, the final titer of TLA reached 3.7 g·L^−1^ using the optimal fermentation medium (Figure 6), which was close to the predicted value 3.6 g·L^−1^. Hence, the validity of this model was verified. Whereas, the titer of TLA reached 0.8 g·L^−1^ using the original fermentation medium. It indicates that an efficient microbial fermentation technology for LA production has been developed using an engineered *C. paspali* strain.

## 4 Conclusion

In this study, an efficient genetic manipulation system, which was based on the protoplast-mediated transformation, was established in the industrial strain *C. paspali*. Based on this, LA and ILA were accumulated in the *ΔlpsB* strain. By single factor optimization, PBD and BBD optimization in shaking flasks, an optimal fermentation medium composition was obtained. The final titer of TLA reached 3.7 g·L^−1^, which was 4.6-fold of that in the initial medium. Our work provides an efficient strategy for LA production, and lays the foundation for its industrial application in the future.

## Data Availability

The original contributions presented in the study are included in the article/Supplementary Material, further inquiries can be directed to the corresponding authors.
