# Supplementary material for: Construction of an efficient Claviceps paspali cell factory for lysergic acid production
Source: Front Bioeng Biotechnol. 2023 Jan 25;10:1093402. doi: 10.3389/fbioe.2022.1093402 (PMC9905238; doi:10.3389/fbioe.2022.1093402)
Supplement: Supplementary file 1 [file DataSheet1.docx]

Supplementary Material

Supplementary Table 1. Primers used in this study.

| Primers | Sequence（5’-3’） |
| --- | --- |
| Up-lpsB-F | ctgtggccaaacttccttcgt |
| Up-lpsB-hph-R | ggtaggccgagaacctcagcaaaggttcgggatcgcaagcgtaaag |
| Down-lpsB-hph-F | cctgggttcgcaaagataattgaaccgtcggcgtgagactcag |
| Down-lpsB-R | gtatggcatatctcgagctcc |
| N-lpsB-F | gaccgatttgtcgctgcgaac |
| N-lpsB-R | ggcactcaattcggcttacag |

Supplementary Table 2. Optimization of regeneration medium with different concentrations of D-sorbitol.

| Concentrations of D-sorbitol in plates | 0 M | 0.2 M | 0.4 M | 0.6 M | 0.8 M |
| --- | --- | --- | --- | --- | --- |
| Average Numbers of clones per plate | 0 | 8 | 30 | 19 | 6 |

Supplementary Table 3. ^1^H and ^13^C NMR data of compounds lysergic acid (LA) and iso-lysergic acid (ILA).

|  | LA | | ILA | |
| --- | --- | --- | --- | --- |
| *No.* | *δ_H_* (*J* in Hz) | *δ_C_* | *δ_H_* (*J* in Hz) | *δ_C_* |
| N_1_-H | 11.69 (s) |  | 11.70 (s, 1H) |  |
| 2 | 7.26 (s) | 120.5, CH | 7.21 (s, overlapped with *Pyridine*) | 120.4, CH |
| 3 |  | 111.0, qC |  | 110.9, qC |
| 4β  4α | 3.64 (dd, *J* = 14.5, 5.6)  2.96-2.89 (m) | 28.3, CH_2_ | 3.57 (dd, *J* = 14.5, 5.6, 1H)  2.89 (ddd, *J* = 14.4, 11.5, 1.8, 1H) | 28.0, CH_2_ |
| 5 | 3.31 (m) | 64.2, CH | 3.33 (m, 1H) | 64.0, CH |
| N_6_-CH_3_ | 2.54 (s) | 43.7, CH_3_ | 2.52 (s, 3H) | 43.9, CH_3_ |
| 7β  7α | 3.54 (dd, *J* = 11.3, 5.0)  2.96-2.89 (m) | 56.4, CH_2_ | 3.61 (dd, *J* = 11.8, 2.9, 1H)  2.79 (dd, *J* = 11.5, 4.2, 1H) | 54.9, CH_2_ |
| 8 | 4.09-4.04 (m, 1H) | 44.4, CH | 3.54-3.50 (m, 1H) | 42.7, CH |
| 9 | 7.18 (brs) | 120.3, CH | 7.01 (d, *J* = 4.8, 1H) | 120.1, CH |
| 10 |  | 137.2, qC |  | 137.4, qC |
| 11 |  | 129.3, qC |  | 129.5, qC |
| 12 | 7.46 (d, *J* = 7.2) | 112.7, CH | 7.43 (d, *J* = 7.2, 1H) | 112.6, CH |
| 13 | 7.31 (t, *J* = 7.6) | 123.6, CH | 7.34 (t, *J* = 7.6) | 123.7, CH |
| 14 | 7.44 (d, *J* = 8.0) | 110.9, CH | 7.45 (d, *J* = 8.0, 1H) | 111.0, CH |
| 15 |  | 135.6, qC |  | 135.7, qC |
| 16 |  | 127.8, qC |  | 127.8, qC |
| 17 |  | 175.4, qC |  | 175.5, qC |


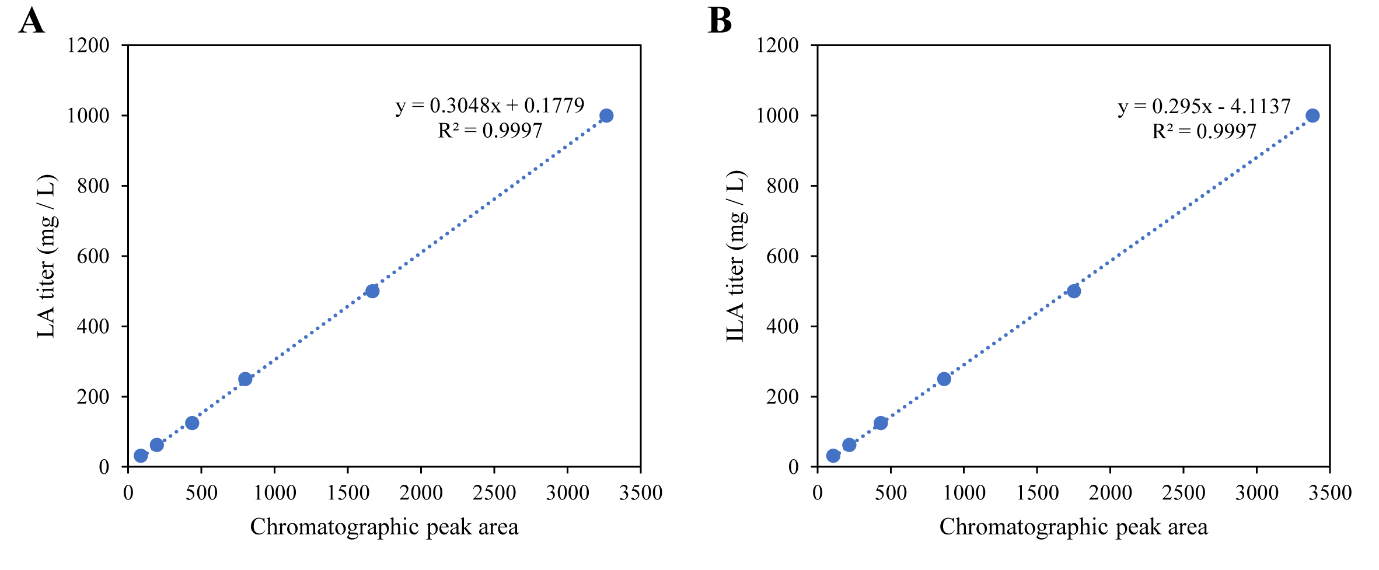


Supplementary Figure 1. The calibration curves for the content of LA (A) and ILA (B).


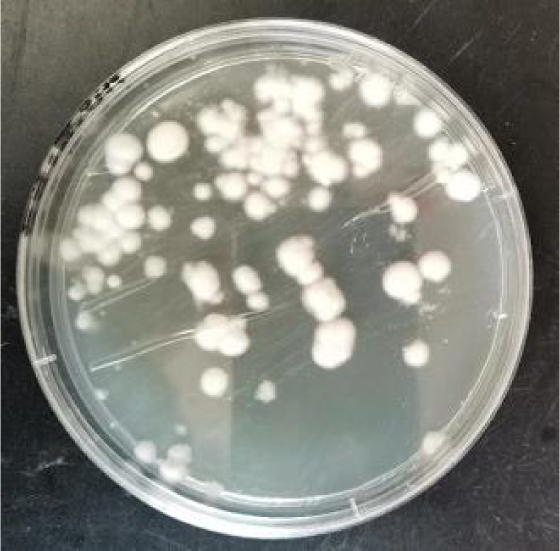


**Supplementary Figure 2.** Original transformation plates.


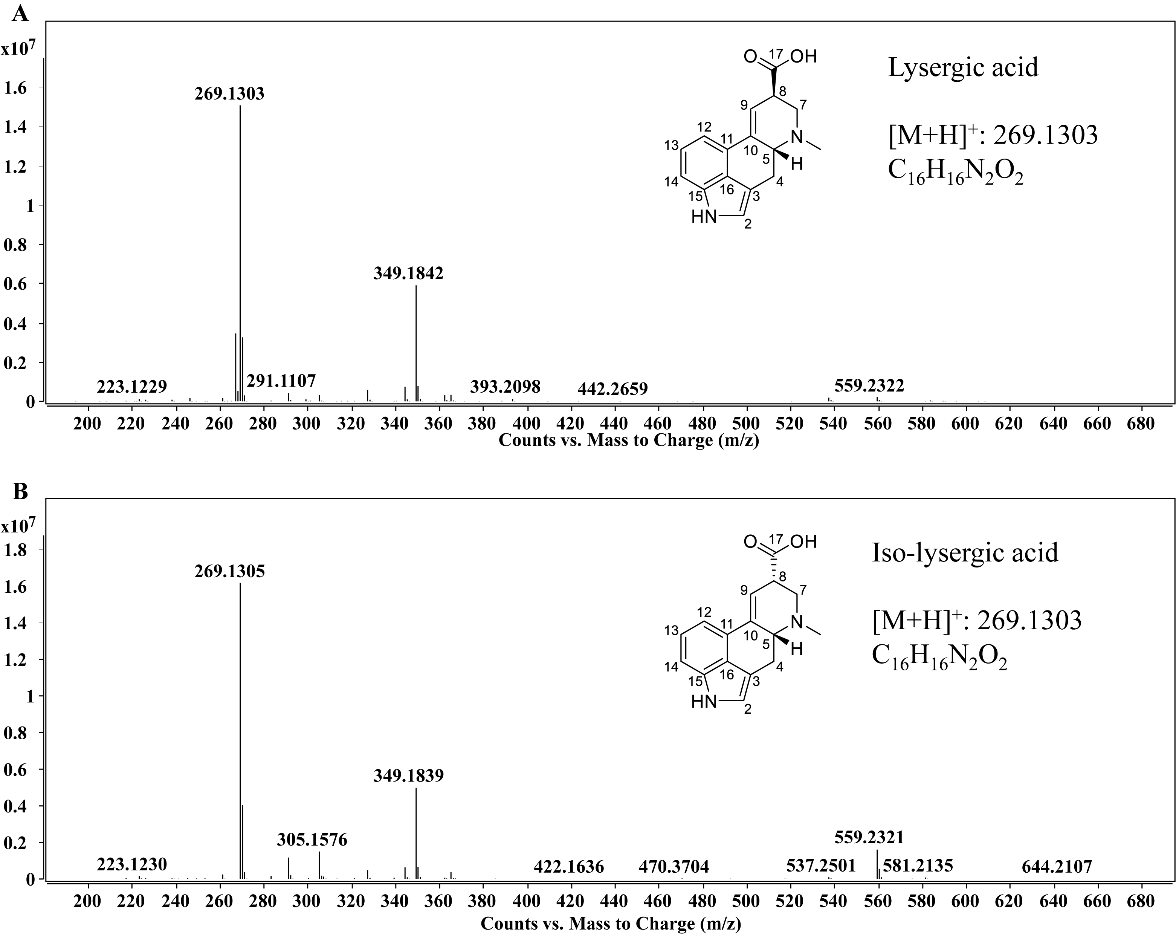


Supplementary Figure 3. LS/MS fragmentation spectra of LA (A) and ILA (B).

**
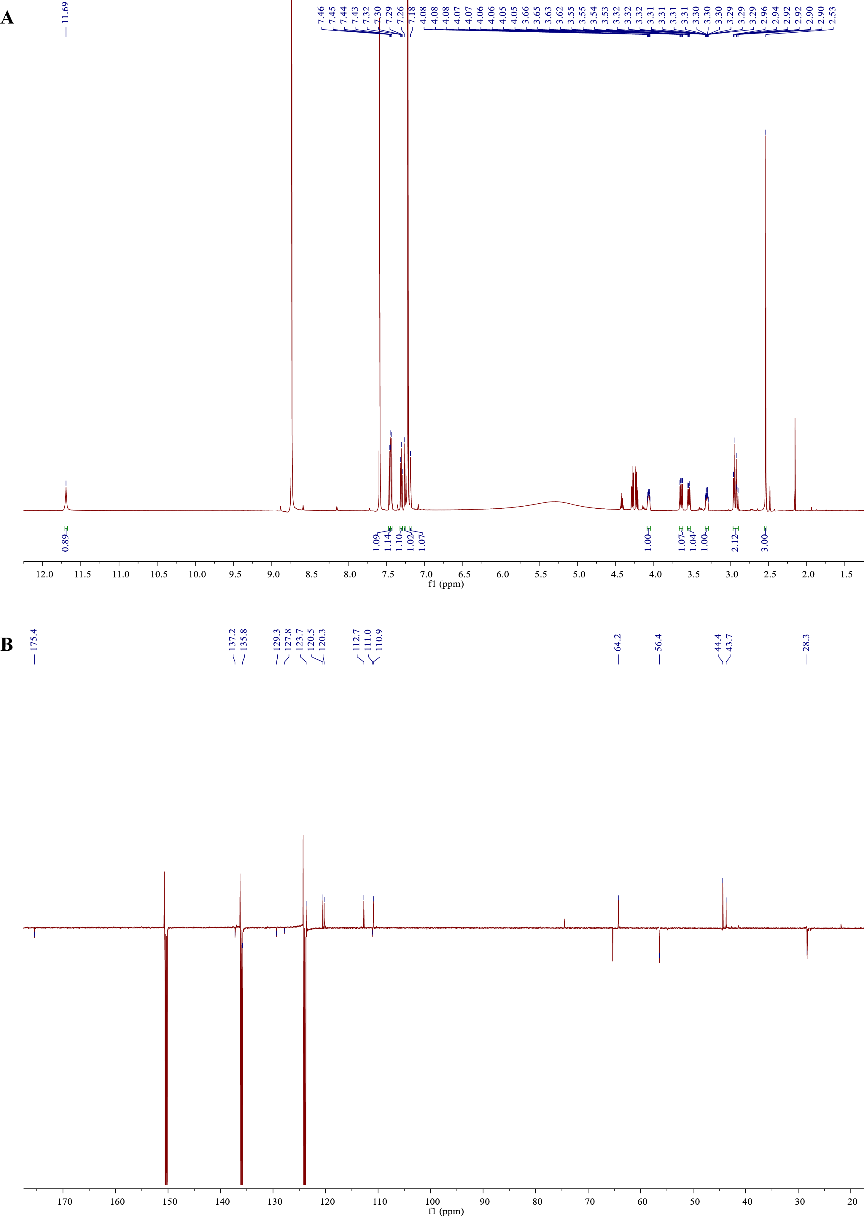
**

Supplementary Figure 4. NMR data of purified LA.

(A) ^1^H NMR spectrum (600 MHz, Pyridine-*d_5_*); (B) ^13^C NMR spectrum (151 MHz, Pyr).


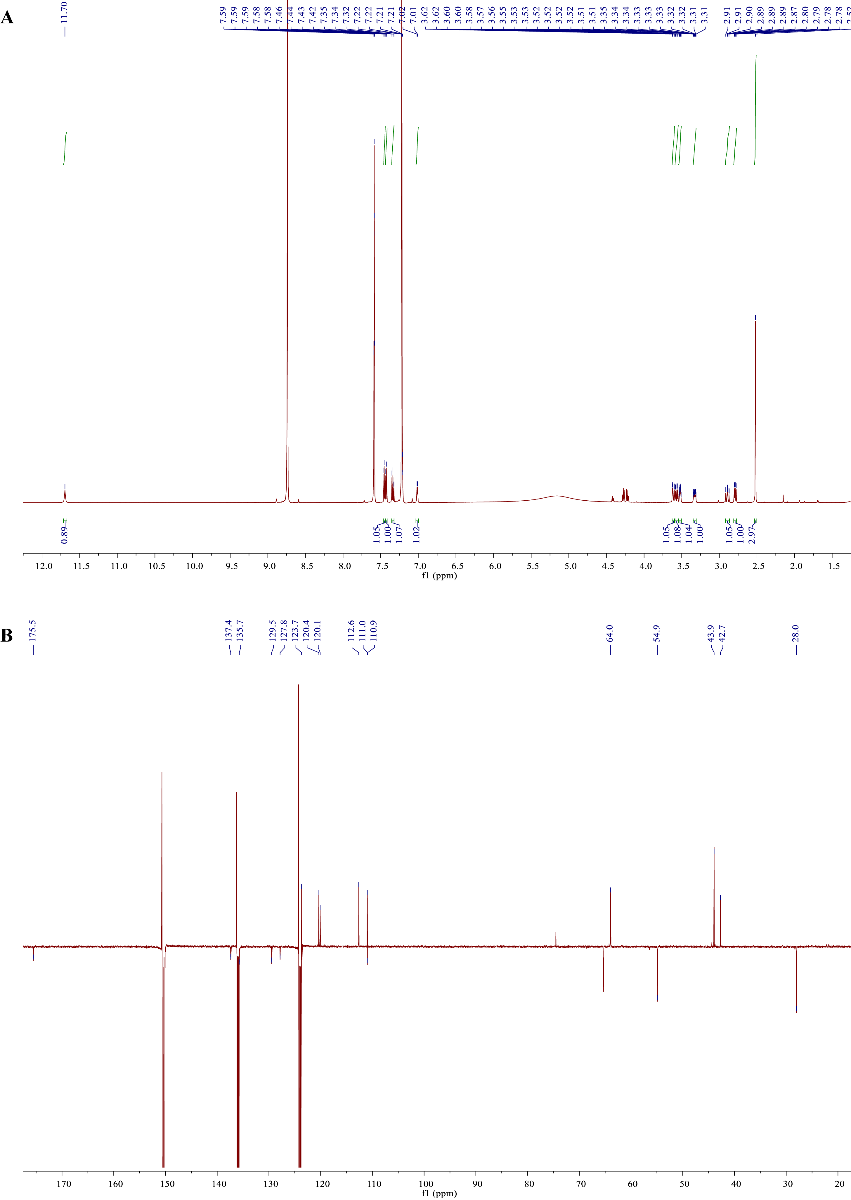


Supplementary Figure 5. NMR data of purified ILA.

1. ^1^H NMR spectrum (600 MHz, Pyridine-*d_5_*); (B) ^13^C NMR spectrum (151 MHz, Pyr).

**References**

Liu, Q., and Jia, Y. (2011). Total synthesis of (+)-lysergic acid. *Org. Lett.* 13 (18), 4810-3. doi: 10.1021/ol2018467

Umezaki, S., Yokoshima, S., and Fukuyama, T. (2013). Total synthesis of lysergic acid. *Org. Lett.* 15 (16), 4230-3. doi: 10.1021/ol4019562
